# A Synopsis of Two Decades of Arthropod Related Research at the Forensic Anthropology Research Facility (FARF), Texas State University (TXST), San Marcos, Texas, USA

**DOI:** 10.3390/insects16090897

**Published:** 2025-08-27

**Authors:** Tennyson B. Nkhoma, Gabriella D. Rakopoulou, Scott H. Fortney, Daniel J. Wescott, Katherine M. Spradley, Ian R. Dadour

**Affiliations:** 1 Entomology and Nematology Department, University of Florida, Gainesville, FL 32611, USA; tennysonb.nkhoma@ufl.edu (T.B.N.); gabriella.rakopoulou1998@gmail.com (G.D.R.); scott.fortney@ufl.edu (S.H.F.); 2 Dermestid Beetle Colony Operations, Cheyenne Mountain Zoo, Colorado Springs, CO 80906, USA; 3 El Paso County Coroner’s Office, Colorado Springs, CO 80906, USA; 4 Department of Anthropology, Forensic Anthropology Center at Texas State, Texas State University, San Marcos, TX 78666, USA; dwescott@txstate.edu (D.J.W.); mks@txstate.edu (K.M.S.); 5 School of Medical, Molecular & Forensic Sciences, Murdoch University, Perth, WA 6150, Australia; 6 Source Certain, Perth, WA 6947, Australia

**Keywords:** forensic entomology, forensic acarology, arthropod-related research, anthropology, forensic science, human taphonomy facilities, human decomposition, postmortem interval

## Abstract

The Forensic Anthropology Research Facility (FARF) at Texas State University (TXST), San Marcos, TX, USA, is a leading human taphonomy facility (HTF), dedicated to advancing forensic science through the study of human decomposition. This systematic review synthesizes 15 scholarly outputs comprising 7 peer-reviewed journal articles and 8 dissertations centered on arthropod-associated research undertaken at FARF, with particular emphasis on its contribution to forensic entomology. The analyzed body of literature is organized into six overarching thematic domains: (1): refining postmortem interval (PMI) estimation; (2): developmental biology of forensic arthropods; (3): arthropod behavior and forensic implications; (4): Taxonomy and systematics; (5): microbial–arthropod interactions; and (6): forensic decomposition scenarios with arthropod involvement. Key contributions from these studies include refined methodologies for PMI estimation, the systematic revision of forensically relevant arthropods and identification of accidental arthropod activity. Additionally, studies at FARF have incorporated interdisciplinary approaches bridging entomology, microbiology and ecology. The semi-arid, subtropical environment and large open natural range of FARF provides some unique regional and specific insights concerning decomposition. This entomological review on FARF is the first to be completed concerning any HTF and adds to the knowledge of forensic evidence involving arthropods.

## Simple Summary

The present review summarizes the arthropod-related research conducted at the Forensic Anthropology Research Facility (FARF), which operates under the auspices of Texas State University (TXST). It draws on entomology-related research from the establishment of FARF in 2008 to the present. FARF serves as a key site for investigating postmortem biological processes under natural conditions and is the largest of 15 human decomposition research facilities, with 12 located in the United States of America and 3 internationally. While these facilities focus on various aspects of taphonomy, they are primarily operated by forensic anthropologists. Forensic entomology, which examines the role of insects and other arthropods in legal investigations, has been studied directly and indirectly at the FARF. The results documented a wide array of arthropod taxa on human remains, with particular attention given to necrophagous insect groups such as blow flies (Diptera: Calliphoridae) and beetles (Coleoptera), which have been utilized in some studies to aid in the estimation of the time since death (TSD). Further studies at FARF have explored the interactions between arthropods and microbial communities to better understand their roles in the decomposition process. Given the subtropical climate of the region, the activity patterns of these organisms are closely linked to abiotic variables such as temperature (T) and relative humidity (RH). Although these findings show promise for improving forensic methodologies, they are accompanied by methodological challenges and contextual limitations that merit further investigation. The central aim of this review is to promote greater involvement of forensic entomology in human taphonomic facilities (HTFs), to support law enforcement and enhance the resolution of forensic casework.

## 1. Introduction

### 1.1. Introduction to Human Taphonomy Facilities (HTFs)

Human Taphonomy Facilities (HTFs), colloquially referred to as “body farms” [1], function as specialized outdoor research centers devoted to the scientific examination of human decomposition [2,3]. Prior to the establishment of such facilities, forensic pathologists and anthropologists relied on laboratory and animal experiments to construct a human postmortem interval (PMI) [4]. Outside such facilities the animal model remains the sole research analog. Contemporary research conducted at HTFs has provided a vanguard for enhancing the accuracy of PMI estimations [2,4] and understanding human decomposition in general [2,3,5]. The research conducted in HTFs is an accumulation of data and observations collected by researchers observing how bodies decompose under various scenarios. [6]. The data enhances and builds upon research using animal models [7,8,9]. These include intrinsic factors such as body size, body composition (e.g., fat and muscle content), age, cause of death and pre-existing medical conditions [10,11]. Extrinsic factors including temperature, humidity, soil composition, exposure to sunlight or shade, clothing, local fauna, microbial activity, vegetation and the extent of exposure to insects and scavengers [12,13,14,15,16,17,18] can also be measured. The data generated from such research in HTFs are essential for the judiciary to understand the process of decomposition [1,2,19,20,21,22,23,24,25]. As well, the benefits of HTFs for the forensic community extend beyond research, offering opportunities for training police, military and law enforcement personnel, and research students in the recovery of human remains [26].

### 1.2. The Establishment and Global Expansion of HTFs

The first HTF facility commenced operation in 1981 with the establishment of the Forensic Anthropology Facility (ARF) at the University of Tennessee, Knoxville, USA [14] and is overseen by the Anthropology Department at the University of Tennessee. The facility was initially designed for systematic observation and documentation of decomposition, to improve PMI estimations in forensic cases and to ensure the secure handling of evidence in forensic investigations [27]. Research institutes with established body donation programs aimed specifically at decomposition related research are becoming more prevalent and can now be found globally [3] emphasizing the necessity for region specific data to improve forensic methodologies and outcomes [1,28].

The number of HTFs has increased over the last two decades as a consequence of universities interacting with communities in awareness campaigns. The establishment of these HTFs has become more acceptable to the general public as research facilities deliver an understanding about forensic science. This has become evident due to a willingness of individuals to donate themselves following death for experimental research [4]. As of 2025, at the time of publication, there are 15 operational HTFs, with 12 located in the United States of America and 1 facility each in Australia, the Netherlands and Canada [5,29,30]. Table 1 provides an overview of HTFs worldwide. The body of research produced by these facilities plays an essential role in advancing taphonomy and anthropology as well as the many other forensic aspects associated with these disciplines [24,31,32,33,34].

### 1.3. The Forensic Anthropology Research Facility (FARF) at TXST, San Marcos, TX, USA

**Figure 1:**
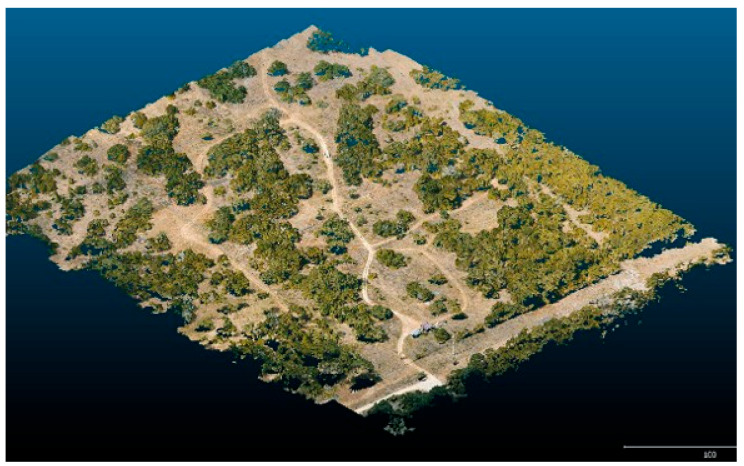
Aerial 3D reconstruction of the northern area of FARF depicting the basic layout and vegetation.

The Forensic Anthropology Research Facility (FARF) (Figure 1) at Texas State University (TXST), is an anthropology laboratory conducting both surface and burial human decomposition studies [35]. The facility was established in 2008 [31] and is located approximately 11.3 km from the TXST main campus [36]. FARF, recognized as the largest human decomposition research site globally, encompasses 10.52 fenced hectares within the 1416-hectare Freeman Ranch in San Marcos, TX. Of this total, approximately 2.02 hectares are explicitly designated for taphonomic research, allowing for controlled studies on human decomposition under natural environmental conditions [37]. The purpose of this facility is to address the need for decomposition research specific to central Texas [10] which experiences a humid subtropical climate interrupted with drought and semi-arid conditions [38]. Weather conditions at FARF include an average humidity of ~77%, an annual precipitation of ~857 mm, a mean annual temperature of ~19.4 °C and an average wind speed of ~3.6 mph [31,38]. The topography at FARF is predominantly flat, with elevations ranging from approximately 204.2 to 286.5 m [31] and comprises perennial grasslands interspersed with natural vegetation of Ashe juniper (*Juniperus ashei* J. Buchholz), live oak (*Quercus fusiformis* Small), prickly pear cactus (*Opuntia* spp. Mill.), elm (*Ulmus* spp. L.). The facility is established on stony, clay-rich soils derived from eroded limestone [39], with shallow bedrock and significant gravel and cobble content [31,36]. The local fauna includes coyotes, foxes, raccoons, skunks and rattlesnakes, which have been recorded within the facility despite the presence of fences designed to deter animal intruders and scavengers [36]. Decomposition at FARF typically results in mummification, with mold observed on human remains regardless of whether the remains are exposed or covered [36].

FARF is an interdisciplinary facility, with integrative and collaborative research programs and encompasses many sciences, such as forensic entomology [40] forensic acarology [41] geology [42], microbiology [43] pathology and medicine [44], ecology [45] botany [46], chemistry [47]. Research conducted at FARF generally focuses on the interaction between environmental factors and human decomposition [10]. In addition, FARF facilitates scientific conferences, specialized workshops (e.g., forensic entomology (https://www.txst.edu/anthropology/facts/workshops/taphonomy.html, accessed on 10 August 2025), and outreach initiatives, while also providing advanced training for law enforcement personnel and training human remains detection dogs [48,49] and includes burial, surface and caged placement scenarios depending on donor consent and research protocols [37].

While the current research focus of FARF is on human cadavers [43,50,51,52], select studies involving animal remains are also conducted to support comparative research [36]. Research initiatives at FARF include examining the effects of vulture scavenging on human remains [48,52] and studying the decomposition of deceased individuals while attempting to cross the U.S./Mexico border [53]. The Forensic Anthropology Center at Texas State (FACTS), which encompasses FARF, was established in 2006 and houses the Osteological Research and Processing Lab (ORPL), responsible for the administration of forensic anthropological casework, the cleaning of any soft tissue remnants and the processing of donated skeletal remains [48]. FACTS also oversees the Grady Early Forensic Anthropology Research Laboratory (GEFARL), established in 2017, which maintains the Texas State Donated Skeletal Collection (TXSTDSC). All forensic data is deposited into the Forensic Data Bank, which supports forensic casework and broadens the applicability of the facility beyond decomposition research. This multipurpose facility also houses an animal skeletal collection [36]. An overview of the practices and processes carried out by FACTS are detailed in the review by Gotcha et al. [37].

### 1.4. Animal Analogs in Forensic Taphonomy

Prior to 1981 and HTFs, the only decomposition studies that were able to be conducted were on animal models. Since the pioneering studies by Bornemissza [54] on guinea pigs *Cavia porcellus* (Linnaeus) and by Payne [55], Payne et al. [7] and Payne and King [56] on domestic pigs *Sus scrofa domesticus* (Linnaeus), animal remains, typically pigs, have been the mainstream organism in taphonomic research globally [25,57,58,59,60,61,62]. This is due to pigs having anatomical and physiological similarities to humans [63], including organ structure, fat distribution, omnivorous diet, and minimal body hair [64,65,66,67,68]. Animal models as part of well-designed experiments offer one main advantage and that is ensuring that outcomes are statistically valid [26]. Forensic scientists, including entomologists, have often extrapolated that findings derived from non-human carcass studies are applicable to human remains [62]. The porcine model has undoubtedly contributed greatly to this understanding [69,70,71,72,73,74]. Despite this, the use of non–human models in place of human cadavers for baseline forensic studies now face scrutiny and some indifference regarding their scientific validity in the courtroom [66,67].

HTFs have played a pivotal role in addressing these concerns by enabling comparative entomological studies between human and non–human models [62,75,76,77]. The ability of HTFs to facilitate these studies under varying environmental conditions is crucial for refining forensic entomology methodologies and verifying the reliability of arthropod evidence in investigative settings.

### 1.5. Arthropod-Related Studies in Forensic Science

Colonization of the corpse by extrinsic organisms may begin within hours [68], minutes [78] or even seconds following death [79]. Of particular significance are arthropods that have been recorded as important forensic indicators [80]. Arthropods provide critical insights into the timing and progression of decomposition [81]. Forensic entomology and forensic acarology are complementary disciplines within forensic taphonomy and are integral to the PMI estimation. Many arthropod taxa, both terrestrial and aquatic, have been consolidated into the discipline of forensic entomology. This is a consequence of medical examiners and the judiciary not understanding how this evidence should be reconciled. Therefore, these groups in this review will be referred to collectively as forensic entomology [82]. Necrophagous insects, particularly Diptera (true flies) and Coleoptera (beetles), are the predominant taxa colonizing remains [83]. Their immature stages are typically used to determine the minimum postmortem interval (PMI_min_) [81,84].

Forensic entomology can be used to provide information concerning whether remains have been moved or concealed postmortem, the season and geographical context of death [85], the timing of dismemberment [86] and the identification of trauma locations [87], indicating potential weapons involved [88]. Other studies have used insects to detect the presence of drugs or poisons [Entomotoxicology] [89,90] and determine submersion intervals [91], associate suspects with crime scenes [81], aid in sexual assault cases and facilitate suspect identification through the association of DNA evidence [92,93,94]. Insects can also serve as indicators in cases involving human or animal abuse and neglect [81] and poaching [95].

Forensic acarology primarily focuses on the use of mites for PMI estimation. Mites, owing to their high diversity, widespread distribution and abundance make them significant contributors regarding the movement or relocation of remains and may link suspects to crime scenes [96]. Their accurate interpretation of arthropod occurrence, behavior and development under varying environmental conditions is also vital, as factors such as temperature and humidity significantly influence these processes [1] underscoring the importance of region-specific arthropod data to improve casework accuracy.

Despite the expansion of HTFs, skepticism persists among some forensic scientists and the judiciary regarding the contributions of research published by these facilities, with critiques often focusing on limitations such as small sample sizes with insufficient methodological rigor [96,97]. As a result, the debate on using human and non-human models has become a paradoxical situation about whether animal models actually represent human decomposition. This perspective highlights the need for reviews that assess the contributions and methodological approaches of HTFs to enhance transparency and credibility of these human studies within forensic science. To conclude, the overarching aim of this review is to synthesize the current state of arthropod-related research conducted at FARF and examine its contribution to forensic science, while identifying potential areas and/or strategies for future exploration.

## 2. Materials and Methods

### 2.1. Systematic Review Framework

A systematic literature review was conducted in accordance with the Preferred Reporting Items for Systematic Reviews and Meta Analyses (PRISMA) reporting guidelines [98] to ensure methodological rigor, transparency and reproducibility. The present review examined peer reviewed journal articles and dissertations pertaining to arthropod–related forensic research, including studies in forensic entomology and acarology, conducted wholly or partially at FARF.

### 2.2. Published Literature Search Strategy

The systematic literature search encompassed the entire period from when FARF was first established until 2025, and was performed across multiple academic databases, including PubMed, Web of Science, Scopus, Google Scholar and ProQuest. Relevant studies were identified using a predefined set of search terms, which included: FARF, forensic entomology, postmortem interval estimation, forensic acarology, taxa, insects, mites, arthropods, Texas State University, human decomposition, flies, beetles and entomological evidence. Citation tracking was also employed to locate additional relevant studies through references cited in the selected articles. The initial search was undertaken in November 2024, with periodic updates maintained through July 2025 to contribute to the development of the final systematic review.

### 2.3. Incorporation of Dissertations

In order to ensure a comprehensive and representative synthesis of arthropod focused research undertaken at the FARF, unpublished academic dissertations pertaining to forensic entomology and acarology were included in the present review. These documents, which reflect original investigations conducted wholly or partially at FARF, were subjected to the same inclusion and exclusion criteria established for peer-reviewed literature. Complementing dissertations retrieved through publicly accessible databases, authorization was granted by TXST to access additional academic works conducted at the facility, thereby permitting their systematic evaluation and inclusion in the overall analysis.

### 2.4. Selection Criteria

#### 2.4.1. Inclusion Criteria

Eligibility for inclusion was restricted to studies that investigated taxa ecologically or forensically linked to human decomposition mostly conducted within FARF. These are grounded in experimental methodologies or systematic data collection and contributed directly to forensic entomology either through applied arthropod-related analyses or through the documentation of any arthropod taxa observed. Considered sources included peer reviewed journal articles and academic dissertations that met these criteria, which ensured a comprehensive representation of relevant research. Additional relevant articles that satisfied the inclusion criteria were identified from the references cited in the original articles retrieved. The search results were exported in plain text format, with the full records and corresponding cited references for each document subsequently collected. Those records included authorship, year of publication, type of record (peer reviewed original journal article or academic dissertation), the results of any arthropod-related research, and the presence and identification of arthropod taxa.

#### 2.4.2. Exclusion Criteria

The search exclusionary measures omitted specific document types, including proceedings papers, editorial materials and conference abstracts. Studies that were not directly related to the scope of forensic arthropod research conducted at FARF were eliminated following a preliminary screening of titles and abstracts. Other published articles or dissertations were excluded if they did not demonstrate a direct linkage to the FARF. These exclusion criteria and their associated parameters were established to ensure that only relevant studies were retained for analysis of the final dataset.

#### 2.4.3. Data Extraction and Thematic Classification

Relevant data were systematically extracted from the selected studies, encompassing study objectives, methodologies, principal findings and their broader implications for forensic entomology/arthropology. The literature was subsequently organized into distinct thematic categories that encapsulate the scope of forensic arthropod research conducted at FARF. 

#### 2.4.4. Screening and Selection Process

The screening process was conducted in two stages to ensure the inclusion of studies directly relevant to forensic arthropod research at FARF. The initial screening involved an assessment of studies based on their examination of forensic arthropod taxa, with a particular focus on species within the Class Insecta and Arachnida in the context of human decomposition at the facility. In the subsequent review, only studies explicitly addressing the above themes were retained for analysis. Given the qualitative and summative nature of this systematic review, statistical analyses were not required.

## 3. Results

### 3.1. Review of Literature

**Figure 2:**
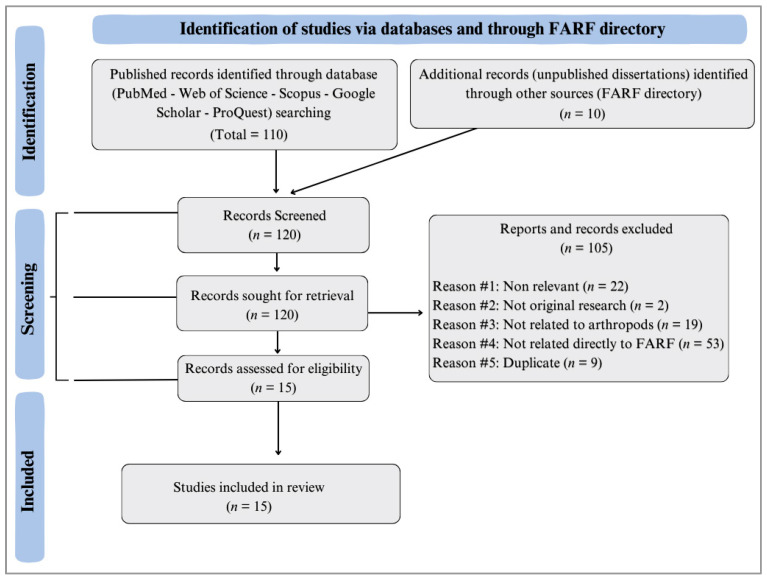
Systematic review selection process: descriptive PRISMA flow diagram of published articles and dissertations screened and included in the present review.

A total of 110 published articles and 10 dissertations were screened. After this preliminary assessment, 7 published original articles and 8 dissertations met the required criteria for inclusion (Table 2). Consequently, a total of 15 scholarly outputs were included in this review (Figure 2).

The retrieved published articles and dissertations are summarized in Table 2, organized by author(s), year of publication, type of work (published record or dissertation), and major findings and contributions, with published articles presented first, followed by dissertations, both arranged in chronological order based on year of publication.

### 3.2. Published Research Involving Arthropods at the Forensic Anthropology Research Facility (FARF), TX, USA

Concerning the published literature originating from FARF, 7 studies were identified, each contributing directly to forensic entomology, acarology and insect/decomposition ecology. Table 3 presents a chronological compilation of taxa documented in research conducted at FARF between 2007 and 2023, as reported in both peer reviewed publications and academic dissertations. The entries are arranged by year of record and include the literature source (published record or dissertation), scientific name (taxon), common name or ecological descriptor, higher level taxonomic classification and family designation. These investigations all addressed the specified themes (Table 4). These themes included (1) refining PMI estimations through arthropods dynamics; (2) Developmental biology of forensically relevant insects; (3) arthropod behavior and forensic implications; (4) taxonomy and systematics; (5) microbial arthropod interactions; and (6) forensic decomposition scenarios with arthropod involvement.

#### 3.2.1. Development and Validation of a New Technique for Estimating a Minimum Postmortem Interval Using Adult Blow Fly (Diptera: Calliphoridae) Carcass Attendance

Mohr and Tomberlin [100] developed and validated a methodological framework for estimating the PMI_min_ using adult blow fly (Diptera: Calliphoridae) carcass attendance. The study examined four blow fly species including *C. macellaria*, *C. rufifacies*, *P. regina*, and *C. vicina*, across seasonal conditions, assessing species specific arrival patterns, sex-based attendance differences, and oocyte development in relation to carcass exposure. Research results indicated that summer active species arrived within 4–12 h, while winter active species exhibited delays of up to 48 h. The technique and having all the information at hand, this study, when validated against forensic case simulations involving human and swine remains, successfully predicted time of placement (TOP) in six of seven trials. These findings underscore the potential forensic utility of adult blow fly presence in PMI_min_ estimation, a metric that has traditionally relied on larval development.

#### 3.2.2. Redescription of *Myianoetus muscarum* (Acari: Histiostomatidae) Associated with Human Remains in Texas, USA, with Designation of a Neotype from Western Europe

O’Connor et al. [41] contributed to forensic acarology by conducting a taxonomic revision of *M. muscarum,* a mite species associated with human decomposition. The study addressed long standing taxonomic ambiguities by reviewing historical specimens from North America and Europe, with the designation of a neotype from Belgium. Specimens collected from human remains at FARF were analyzed morphologically and systematically to clarify species distinctions. Given the growing forensic interest in mites as postmortem indicators [95], the study provides a revised diagnostic framework for the identification of *M. muscarum*, facilitating its forensic application in cases where mite presence may inform decomposition timelines and postmortem intervals.

#### 3.2.3. Field Documentation of Unusual Postmortem Arthropod Activity on Human Remains

Pechal et al. [40] documented postmortem activity of arthropods, resulting in observable modifications to human remains. The study examined a case at FARF where an adult *P. haldemani* Orthoptera: Tettigoniidae) and *Armadillidium* cf. *vulgare* (Isopoda: Armadillidiidae) were observed feeding on soft tissue. The arthropod induced markings bore morphological resemblance to antemortem and perimortem wounds, raising concerns about potential misinterpretation in forensic casework. Furthermore, *Solenopsis invicta* Buren (Hymenoptera: Formicidae) (red imported fire ants) were documented constructing structural formations within the marks produced by the katydid feeding activity. This study expanded forensic knowledge of arthropod mediated tissue modification, emphasizing the need to distinguish biological activity from traumatic injury when analyzing postmortem remains.

#### 3.2.4. Temporal and Spatial Impact of Human Cadaver Decomposition on Soil Bacterial and Arthropod Community Structure and Function

Singh et al. [45] investigated the spatiotemporal effects of human decomposition on soil bacterial and arthropod communities, contributing to forensic taphonomy and environmental forensics. The study tracked microbial and arthropod responses at varying distances (0, 1, and 5 m) over a period of 3–732 days postmortem, assessing carbon mineralization, microbial functional shifts, and arthropod diversity. The findings revealed a predictable microbial response to decomposition, with increased abundance of Bacteroidetes and Firmicutes in decomposition affected soil, while other bacterial taxa (Acidobacteria, Chloroflexi, Gemmatimonadetes and Verrucomicrobia) exhibited a reduced prevalence. Arthropod abundance was 15 to 17 times higher at cadaver associated sites compared to control sites, yet arthropod community composition showed no correlation with microbial activity. Precipitation was identified as a key environmental factor influencing bacterial succession and microbial function, whereas arthropod assemblages remained temporally stable. The study provides foundational data for the ecological characterization of carrion decomposition, highlighting long term environmental impacts that may inform forensic applications in field investigations.

#### 3.2.5. Evaluation of Development Datasets for *Hermetia illucens* (L.) (Diptera: Stratiomyidae) for Estimating the Time of Placement of Human and Swine Remains in Texas, USA

Cuttiford et al. [102] assessed the forensic applicability of developmental datasets for *H. illucens* (Diptera: Stratiomyidae), a species of forensic significance. The study tested multiple datasets for their accuracy and precision in estimating the time of placement (TOP) in cases involving five human and three swine remains across two locations in Texas (San Marcos, TX, USA and College Station, TX, USA). The findings demonstrated that only one dataset reliably estimated the TOP within a one-day margin of error in 52% (prepupae) to 75% (eclosion to adult) of cases. Furthermore, the study examined the pre colonization interval (PCI), a variable often overlooked in PMI estimation. While *H. illucens* was observed colonizing remains within six days postmortem, the untested assumption of a prolonged PCI introduced variability in accuracy. The study underscores the necessity for standardized, forensic specific developmental datasets for *H. illucens*, as well as improved understanding of resource utilization timelines for forensic entomological casework.

#### 3.2.6. Developmental Variation Among *Cochliomyia macellaria* Fabricius (Diptera: Calliphoridae) Populations from Three Ecoregions of Texas, USA

Owings et al. [99] investigated the impact of genetic variation and environmental conditions on the development of *C. macellaria*, a blow fly species with important forensic applications. Using specimens from three geographically distinct strains collected from human remains, the study reared these populations under two temperature regimes (21 °C and 31 °C) over two years. Results indicated significant genetic differences among strains, with developmental time variability ranging from 2% to 51%, the latter being particularly pronounced under higher temperature conditions. The study found that while immature stages were influenced solely by the environment, the pupal stage exhibited genotype by environment interactions. These results highlight the complex interplay between genetic and environmental factors in influencing developmental traits such as time as well as maggot and pupal mass.

#### 3.2.7. Effect of Intraspecific Larval Aggregation and Diet Type on Life History Traits of *Dermestes maculatus* and *Dermestes caninus* (Coleoptera: Dermestidae): Species of Forensic Importance

Corrêa et al. [101] investigated the type of diet and larval density and how it affects the life history traits of two dermestid beetle species of *D. maculatus*, and *D. caninus*, both of which are of forensic significance. In the study, adult specimens of the two species were collected from human remains and reared under a controlled environment of 27 °C on two diets, dried pork loin or dry dog food. This study revealed that diet significantly influences both larval development and survival. Notably, pork fed larvae exhibited faster growth and greater size than dog food fed ones and specifically *D. maculatus* and *D. caninus* larvae fed on pork were 1.7 and 1.1 times heavier than those on dog food, respectively. Furthermore, this study found that the number of larvae at a given time did not affect the developmental time, length and weight of either species.

#### 3.2.8. Effect of Larval Secretions and Excretion on Selection of Food Source by *Dermestes maculatus* DeGeer

George [106] investigated the influence of larval secretion and excretions on the resource selection of the adult stage of *D. maculatus* which are of significance in forensic entomology. In this study, adult beetles, collected from FARF, were reared under laboratory conditions and used in a Y tube behavioral assay. The study compared the larval and adult beetle preference between dog food treated with larval secretion/excretion (SE) products and untreated dog food (with distilled water only). Pilot studies revealed that the response of beetles increases with age with approximately 60% of beetles aged two weeks or older selected the SE resource. Contrary to the hypothesis that adult beetles would prefer food untreated with SE, the results indicated that they had a slight preference for SE-treated food. The fact that 57.1% of beetles in the second pilot study responded to the SE treated resource, suggests that larval SE products may serve as attractants, potentially signaling a supportive environment for aggregation in beetles.

#### 3.2.9. The Impact of Fat Mass on Decomposition Rate and Postmortem Interval Estimation

Giacomello [109] investigated the influence of body fat content on the rate of human decomposition and how that affects the estimation of PMI. To understand this, the study monitored decomposition of 25 donors (16 females and 9 males) across Body Mass Index (BMI) categories which ranged from underweight (<18.5) to obese class III (>40). The decomposition process was scored using Total Body Score (TBS) and Accumulated Degree Days (ADD) to compare the amount of time that was required to reach the skeletonization of the body trunk. Blow fly larvae were also used to understand their feeding preference based on fat mass. Additionally, subcutaneous fat was collected from 9 donors (8 females and 1 male) to measure BMI. The results from this study indicated no significance difference in early to advanced decomposition across BMI categories (*p* = 0.131), although a trend toward significance emerged when comparing obese who had longer time to reach skeletonization as compared to non-obese individuals (*p* = 0.096). BMI was found to be inaccurate when estimating the fat mass of donors across the obese classes or between obese men and women. Additionally, results suggested that larvae of blow flies do not prefer high fat content; however, their preference did not significantly affect the rate at which bodies decompose.

#### 3.2.10. A Study on the Rate of Decomposition of Carrion in Closed Containers Placed in Shaded Areas Outdoors in Central Texas

Hyder [103] investigated the rate of decomposition of carrion in closed containers placed in an outdoor area with tree shade in central Texas, simulating homicide scenarios where human remains are placed in makeshift coffins. This study used ten feral hogs (*S. scrofa*) in which one served as control and exposed to the natural environment. The goal of the study was to understand how closed containers combined with extrinsic factors such as temperature, humidity and limited insect activity affected the rate of decomposition. The study found that the decomposition process in containers was significantly slower, taking approximately 4 times longer than the control which fully skeletonized in 29 days. The containers exhibited prolonged early decomposition stages with minimal insect activity and greater retention of moisture. These findings highlight the contribution that closed containers have to the decomposition process and underscores the importance of these restricted access environments when estimating the PMI.

#### 3.2.11. Assessing the Effects of Clothing on Human Decomposition Rates in Central Texas

Phalen [104] explored the impact of clothing on the rate and pattern of decomposition of a decomposing body. The study examined three clothed human remains and compared the results to longitudinal data on unclothed remains. The clothed individuals were dressed in cotton polyester blended sweatpants and sweatshirts modified with velcro slits to allow for the observation of limbs. The results indicated that clothed remains exhibited accelerated mummification compared to unclothed remains with mean ADD values generally lower than unclothed control at the point of mummification or skeletonization. The results from this study using a Mann–Whitney U test statistical analysis showed no significant difference in decomposition rates between clothed and unclothed remains. However, increased insect activity was noted and prolonged moist decomposition in clothed remains, preventing mummification which accelerated decomposition. This suggests that clothing may provide a sheltered environment for larvae which may potentially affect the estimation of PMI. During the decomposition study insects such as *C. rufifacies* and *C. macellaria* were collected and identified (Table 3).

#### 3.2.12. Examining the Effect of the Pre-Colonization Interval of Insect Scavengers on Human Decomposition Rates in Central Texas

Young [110] studied the effect of PCI of necrophagous insects on human decomposition. This study used 16 adult human donors who were placed in FARF between June and October 2022. In this research critical factors affecting insect arrival time and the presence of larval masses were examined using the TBS and ADD to quantify the progression of the decomposition process. The donors were divided into morning and afternoon placement groups to assess the impact of ambient temperature. The demographic variables of doners were also analyzed including the biological sex, age, weight and autopsy status. Key findings from this study revealed that weight of the cadaver significantly influenced insect arrival time, and cadavers that were lighter were colonized earlier. Larval masses, as observed on 11 donors, were consistently evident on cadavers that had undergone autopsy between 240.17 and 689 ADD, primarily during the bloating and active decay stages. No significant differences were found concerning other variables including the time of placement, fly genera [*Lucilia* spp. (Diptera: Calliphoridae) or *Sarcophaga* spp. (Diptera: Sarcophagidae)] or month of placement. This study suggests that body weight and postmortem damage such as autopsy incisions may impact insect activities which underscore their importance in PMI estimations. Forensically significant insects including *Sarcophaga* spp., *Lucilia* spp. and *Solenopsis* spp. (Hymenoptera: Formicidae) were collected and identified (Table 3).

#### 3.2.13. Differential Decomposition of Human Remains in Shallow Burials in the Humid Subtropical Environment of Central Texas

Spaulding [108] examined the decomposition of human remains in shallow burials approximately 70–75 cm deep. The study utilized 6 human donors who were not autopsied and were unclothed to assess the decomposition rate, patterns and variability compared to remains placed on the surface. The TBS and ADD were calculated using the Megyesi et al. [111] index with temperature data collected from probes within the graves and ambient temperature from a weather station. Burials ranged from 38 to 159 days and their exhumations consistently revealed desiccation, and some skeletonization and adipocere formation. Insect activity which consisted of phoretic mites, ants, black soldier flies and unidentified larvae were observed in one grave (Table 3). Statistical analysis revealed a weak correlation between the predicted ADD (of Megyesi) and the actual ADD air/ADD burial (0.314–0.6) indicating the inaccuracy for buried remains. It also supported the literature in that temperatures in the grave were more stable than surface temperatures [112]. The study also found that buried remains decomposed slower than remains on the surface with TBS underestimating ADD in buried remains.

#### 3.2.14. The Use and Abuse of the Degree Day Concept in Forensic Entomology: Evaluation of *Cochliomyia macelleria* (Fabricius) (Diptera: Calliphoridae) Development Database

Cuttiford [107] explored the reliability of the ADD model for predicting the development of *C. macellaria* which is a forensically significant blow fly. This study achieved this though using two different developmental datasets. Larval samples from 29 sets of human remains at the FARF were used over a three-year period from 2013 to 2016. The larvae samples were analyzed for developmental stage, length and weight to estimate the time of placement (TOP) of remains by comparing predictions against the actual TOP. The findings of this study revealed that the results from only 29 cases out of 80 cases correctly estimated TOP though the overall accuracy was not sufficient for evidential use (Table 2). This study identified significant limitations of the ADD model including the influence of constant or fluctuating temperature assumption and the lack of field validation.

#### 3.2.15. The Effect of Plastic Tarps on the Rate of Human Decomposition During the Spring/Summer in Central Texas

McDaneld [105] investigated the impact of plastic tarpaulins (tarps) on the rate of decomposition of human remains to understand forensic scenarios where bodies are concealed using tarps. The study utilized ten human remains wrapped in plastic tarps and ten others unwrapped as the control all placed in a semi-shaded area. The objective of this study was to assess how tarps affect decomposition by altering environmental factors such as humidity, temperature and insect activity using TBS and ADD based on Megyesi et al. [111] method. The results revealed that remains wrapped in a tarp decomposed significantly faster than those not wrapped in a tarp. This conclusion was discussed in the context of insect activity in remains wrapped in a tarp mainly due to higher moisture retention, resulting in a much more conducive environment for immature necrophagous flies.

## 4. Discussion

The corpus of scientific discoveries emerging from FARF holds significant regional relevance [10] and also contributes to the broader academic discourse within the forensic science community [37]. The impact of FARF is further reflected in its support for interdisciplinary and collaborative research initiatives centered on human decomposition [42,43,44,46,47]. The present review represents the first systematic synthesis of the scholarly output of FARF and includes 15 scholarly outputs (7 peer reviewed articles and 8 dissertations), which focus on research involving forensically important arthropods (Table 4). This review synthesizes research contributions produced at FARF concerning forensic entomology, mostly within the context of human decomposition [37,51,52]. Six thematic areas have been categorically identified (Table 4), advancing the understanding on utilizing insects in PMI estimations, forensically relevant arthropod behavior and taxonomy and ecological interactions.

**Table 4:** A categorical synthesis of arthropod-related research outputs produced at FARF, organized into themes. Studies are classified into six research categories, with corresponding references provided. Cumulative totals are included to indicate the distribution by scholarly format and overall study count. [“NA” = Not Available; indicates that no published record or dissertation was identified for the corresponding category].

Category		Published Records	Dissertations	Total	References
No.	Theme				
1	Refining PMI estimations through arthropod dynamics	2	2	4	[100,102,107,110]
2	Developmental biology of forensically relevant insects	2	NA	2	[99,101]
3	Arthropod behavior and forensic implications	1	1	2	[40,106]
4	Taxonomy and systematics	1	NA	1	[41]
5	Microbial–arthropod interactions	1	NA	1	[45]
6	Forensic decomposition scenarios with arthropod involvement	NA	5	5	[103,104,105,108,109]
**Total**		7	8	15	

### 4.1. Thematic Synthesis of Arthropod–Related Research

As evident from Table 4, the arthropod related studies conducted at FARF encompass a diverse array of research foci, each contributing distinct insights into the discipline of FE. The distribution of these studies across six thematic categories [(1) “Refining PMI estimations through arthropods dynamics”, (2) “Developmental biology of forensically relevant insects”, (3) “Arthropod behavior and forensic implications”, (4) “Taxonomy and Systematics”, (5) “Microbial–arthropod interactions”, (6) “Forensic decomposition scenarios with arthropod involvement”] reveals a window into insect–related processes in human decomposition.

#### 4.1.1. Forensic Decomposition Scenarios with Arthropod Involvement

This is the largest category with the highest number of studies (*n* = 5 dissertations) that examines the decomposition process in relevant scenarios such as those where human bodies are covered, buried, or enclosed remains where arthropods play a significant role. Hyder [103] found that enclosed containers limit insect access to bodies thereby reducing decomposition rate. On the other hand, Phalen [104] and McDaneld [105] noted that bodies covered with cloth or tarpaulins retain moisture which accelerates the activity of insect larvae and subsequently decomposition. Furthermore, mimicking burial scenarios, Spaulding [108] observed that shallow burials reduce insect activity and slow down decomposition process. On other scenarios of obesity, Giacomello [109] found that obese bodies take significantly longer time to skeletonize. These studies underscore the need for practices to align different scenarios because traditional PMI estimations have mostly relied only on environmental data to estimate development times of forensically important arthropods. Other studies have also shown that a complex interaction of intrinsic and extrinsic factors might be unique to the decomposition of each body. For example, a study by Ferreira and Cunha [113] in an Atlantic coastal temperate climate of Lisbon, Portugal, demonstrated that bodies kept under similar environmental conditions but in the same context had different decomposition rates. Another study by Schotsmans et al. [114] on crime scenes in Belgium also concluded that shallow burial leads to desiccation of exposed body parts and adipocere formation, both of which reduced insect activity and slowed decomposition process. Scientists need to be mindful of the pros and cons of such studies, using humans generally with little replication presents as much of a challenge to forensic practice as does the science only relying on environmental data. The resultant knowledge of these studies at FARF underscores the ecological ubiquity of arthropods in postmortem contexts, even in the absence of entomology driven hypotheses or methodologies.

The studies also indicate a recurrent pattern in which arthropods were not the focus of the investigation but were consistently observed and reported in studies examining broader determinants of human decomposition. As such, the documentation of arthropod presence within these studies reflects observational rather than taxonomically driven or experimentally controlled entomological inquiry. This is further corroborated by the data presented in Table 2, which reveals that in the majority of the arthropod-related records documented during these studies, either the scientific names or detailed taxonomic designations of collected specimens are absent. Instead, arthropods are often referred to by broad descriptors (e.g., “larval masses”, “adult flies”) or generalized common names (e.g., “beetles”, “fire ants”), with higher level classification (Order or Family) either unspecified or inconsistently reported. This pattern suggests a lack of systematic entomological identification, but also highlights the incidental, yet acknowledged role of arthropods as agents of decomposition. The findings emphasize the potential for valuable entomological insights to arise even from non-specialist research at FARF and point to the need for interdisciplinary collaboration in postmortem investigations, particularly when arthropod mediated processes can affect the interpretation of human decomposition.

#### 4.1.2. Refining PMI Estimations Through Arthropod Dynamics

This category (*n* = 4; 2 publications and 2 dissertations) consists of studies that investigate PMI estimation using insect evidence while leveraging on the dynamic interactions of arthropod colonization and development (Table 4). Studies such as Mohr and Tomberlin [100] and Cuttiford et al. [102] refined methodologies based on blow fly colonization or black soldier fly developmental data, respectively, while Young [110] highlighted how cadaver characteristics modulate pre–colonization intervals. On the other hand, Cuttiford [107], identified limitations in the use of ADD models in predicting development of *C. macellaria* due to temperature fluctuations. These findings underscore the critical role of arthropod evidence in temporal reconstructions of death but highlights challenges from environmental variability that influence developmental cues in insects. This aligns with findings from research conducted in Australia which showed that fluctuation in temperature conditions contributes to differences in larval development of *L. sericata* [115]. Another study based in South Africa, reinforced that decomposition rates vary with season and climates which undermines the reliability of using TBS and ADD in diverse settings [116]. This shows that forensic scientists need to be careful when using general development data of forensically important insects since life history of insects vary based on environmental conditions. Furthermore, another study conducted in Australia by Griffiths et al. [117] highlighted that at certain temperatures some taxa such as Coleoptera might be more forensically informative in estimating PMI than Diptera. This is because places with higher temperatures and humidity cause rapid decomposition as compared to low temperature and humidity regions [117]. These findings show that even if traditional forensic scientists use dipteran taxa, which are usually early colonizers, their role in decomposition time is not consistent across all climate regions, hence the need to understand insects in unique regions to improve PMI estimation.

#### 4.1.3. Developmental Biology of Forensically Relevant Insects

This category (*n* = 2 publications) comprises the studies by Owings et al. [99] and Corrêa et al. [101], which investigated factors that influence the developmental biology of forensically significant insects, critical for estimating PMI. These studies highlighted how factors such as genetics and environmental aspects such as temperature, larval density and diet contribute to variability in insect development, underpinning the use of generalized insect growth models. For instance, research on *C. macellaria* [99] demonstrates that genetic differences in fly populations across Texas ecoregions interact with environmental conditions, especially higher temperature, complicating PMI estimations. Similarly, a study into *D. maculatus* and *D. caninus* [101] underscores the role of resource availability in shaping developmental outcomes of these insects which might cause challenges when nutritional conditions vary at crime scenes. Therefore, these studies emphasize the need to use region specific developmental datasets that are tailored to the subtropical climate of FARF, where rapid decomposition and high humidity amplify insect variability [110]. These studies align with other studies such as [118] who provide evidence of genetic diversity and how it affects developmental rates in other blow fly species, suggesting broad ramifications for estimating PMI. These contributions are particularly valuable in illustrating the developmental plasticity of forensic insects, thereby reinforcing the importance of localized or context specific developmental datasets in entomological casework.

#### 4.1.4. Arthropod Behavior and Forensic Implications

This category (*n* = 2; 1 publication and 1 dissertation) encompasses studies in which the behavioral interactions of arthropods with decomposing tissue or conspecific cues can present challenges in the interpretation of evidence (Table 4). George [106] demonstrated that intraspecific chemical cues in *D. maculatus* drives aggregation behavior, while Pechal et al. [40] documented that arthropods (e.g., Orthoptera, Isopoda) inflict postmortem modifications resembling trauma risking a misdiagnosis of the cause or manner of death. These studies highlight the need for forensic entomologists to distinguish arthropod induced modifications from true trauma and to be aware of the activity caused by insects when reconstructing postmortem events. Other external studies have highlighted the complexity of insect behavior in decomposition environments and caution against the misinterpretation of entomological artifacts [119,120].

#### 4.1.5. Taxonomy and Systematics

This category (*n* = 1, a publication) is represented by O’Connor et al. [41] (Table 4), who addressed the revision of the mite *M. muscarum* collected from human remains. This contribution reflects the importance of precise species identification in forensic contexts, particularly as new taxa are increasingly recovered in forensic casework [121,122,123,124,125,126,127,128,129,130]. Accurate taxonomic classification enhances the reliability of estimating the PMI by linking specific taxa to distinct decomposition stages especially for micro-arthropods like mites which are often overlooked when conducting forensic work.

#### 4.1.6. Microbial Insect Interactions

This is the final category (*n* = 1) which is represented by a publication produced by Singh et al. [45], in which the authors assessed how microbial and arthropod communities evolve around decomposing human remains over time (Table 2 and Table 4). This interdisciplinary study bridges microbial ecology and entomology, revealing how biotic communities interact or diverge during taphonomic progression. Although this is a very understudied area, forensically significant insects might act as vectors of key bacterial and fungal decomposers who then construct a synergy with insects in speeding up decomposition of the cadaver [131]. Another external study identified microbial genera including *Ignatzshineria, Clostridium* and *Proteus* as closely associated with necrophagous insects [132]. These microbes produce enzymes that help insects in degrading tissues, highlighting the mutualistic relationship that drives decomposition efficiency. Similar studies have also highlighted that these microbes produce volatile organic compounds that also attract insects to the cadavers [133,134]. This study underscores the importance of taking into consideration microbial communities in the estimation of PMI.

Together, these 15 studies produced at FARF reflect some of the roles of arthropods in forensic science and emphasize that a more integrative research culture be followed at this HTF. FARF should support more entomology specific investigations [44] concerned with decomposition studies in which arthropods emerge as ecologically and forensically salient variables [45,103,104]. This would help promote the refinement of forensic methodologies and foster interdisciplinary collaboration.

### 4.2. The Taxa Studied at FARF

At FARF, extensive research has focused on arthropods such as blow flies and beetles, which are integral to estimating PMI (Table 3). However, other arthropod taxa, including mites (Acari) and springtails, remain significantly underrepresented despite their ecological and possible forensic importance [135]. Addressing these gaps is crucial for advancing decomposition studies specific to the unique environmental conditions at FARF.

Mites are particularly relevant to decomposition at FARF. O’Connor et al. [41], has documented species like *M. muscarum* associated with human remains, highlighting their role in decomposition. Furthermore, mites such as those in the families Acaridae and Uropodidae show distinct successional patterns influenced by environmental factors like moisture, temperature and substrate type [45]. These characteristics could refine PMI estimations under the variable climatic conditions at FARF, where environmental factors strongly affect decomposition rates. Mites also play a critical role in nutrient cycling by consuming decomposed organic matter, fungi and microorganisms, integrating forensic entomology into the broader ecological framework of decomposition [135].

Springtails, integral to soil ecosystems, are particularly relevant to the semi-arid environment of FARF. Singh et al. [45] identified species within the family Isotomidae during decomposition processes, where their abundance and diversity increased in later stages. Springtails contribute to the breakdown of soft tissues and detritus, with certain species exhibiting habitat specificity that aligns with high microbial activity [136,137]. This specificity offers potential for these organisms to act as forensic markers for microenvironmental conditions unique to remains placed at FARF.

Despite their relevance, smaller arthropods like mites and springtails are understudied due to methodological challenges. Conventional trapping methods, such as pitfall and bait traps, Berlese funnels and direct soil sampling are less effective for capturing these smaller taxa. Additionally, most mites, including necrophagous species, exhibit a phoretic behavior in which they leverage on species of flies or carrion beetles to reach decomposing carcasses [128,138]. Singh et al. [45] demonstrated the importance of integrating advanced sampling techniques to document diverse arthropod communities, including mites (Acaridae and Uropodidae) and springtails (Isotomidae), during human decomposition.

### 4.3. Comparative Analysis: FARF and Other Human Taphonomy Facilities

The location of FARF offers a unique environment for decomposition studies [139]. The semi-arid, subtropical climate, characterized by high temperatures, variable humidity, and occasional drought, accelerates decomposition during summer due to enhanced microbial activity and insect development [104,110]. These environmental factors create a distinct arthropod succession pattern, influencing PMI estimation and decomposition ecology. The research at FARF aligns with other HTFs in the USA, such as the ARF at the University of Tennessee, through its focus on decomposition, taphonomy and arthropod succession. Both facilities conduct controlled studies to refine postmortem interval estimation techniques using environmental data. However, FARF stands out with its emphasis on the interaction between arthropods, microbial activity which are influenced by the semi-arid, subtropical environment of Central Texas [105,140].

In contrast, international HTFs such as the AFTER facility in Australia focus on decomposition under extreme climates, such as prolonged aridity characterized by extreme heat and low moisture, favoring desert adapted arthropod species like flesh flies (Diptera: Sarcophagidae) [5]. These conditions lead to mummification rather than rapid tissue breakdown, significantly altering arthropod succession. Similarly, HTFs in cold climates, such as FROST in Michigan, USA and REST[ES] in Quebec, Canada, observe prolonged decomposition periods due to freezing, which halts insect activity for extended durations. These facilities instead focus on the roles of scavengers and microbial activity on decomposition [132].

On the other hand, the Netherlands HTF employs advanced molecular techniques for forensic analyses [141]. These methods include environmental DNA (eDNA) sequencing to track microbial and arthropod dynamics under cooler conditions, which differ markedly from subtropical environments. FARF contributes to the global discourse by providing data on decomposition dynamics under subtropical conditions, complementing findings from temperate or arid regions. This unique perspective expands the understanding of how varying environments influence decomposition and arthropod succession patterns [142]. For instance, the findings on Calliphoridae succession patterns during Texas summers contrast sharply with data from colder climates, where blow fly activity is delayed. Expanding cross-regional collaborations can illuminate universal trends and region specific deviations, offering forensic science a robust framework for adapting methodologies to diverse conditions.

### 4.4. Contributions and Limitations

FARF has significantly advanced aspects of forensic science, particularly in forensic anthropology. Infrequently, however, FARF has conducted detailed studies on blow flies and their seasonal variations, enhancing forensic applications in subtropical climates [100,110]. Another key contribution is the identification of new arthropod indicators, such as postmortem feeding behaviors by katydids and isopods, which provide valuable insights into atypical decomposition scenarios [40,140]. Additionally, FARF has made strides in forensic acarology by exploring the roles of mites and other acari in decomposition, particularly in contexts where insect activity is limited by environmental conditions [104,105].

The focus of the facility on key arthropod species sometimes overlooks micro arthropods or less studied taxa due to resource constraints. Furthermore, greater collaboration with international HTFs could enhance comparative studies, providing deeper insights into global decomposition processes. Nonetheless, the research conducted at FARF has significantly contributed to forensic taphonomy by addressing critical gaps in understanding decomposition dynamics utilizing insects and advancing methodologies for forensic investigations.

### 4.5. Methodological Challenges

One of the most significant methodological challenges faced by FARF and other HTFs is the limited availability of donor bodies, which often constrains sample sizes. The reliance on body donation programs and ethical parameters means that the number and demographic diversity of donors can be unpredictable, restricting the ability to design experiments with sufficient statistical power. This is particularly challenging for studies requiring broad representation across environmental conditions or donor characteristics, as smaller sample sizes limit the interpretability of findings [110,140].

Environmental variability also presents a major challenge to research conducted at FARF. The semi-arid, subtropical climate of Central Texas is characterized by extreme seasonal fluctuations in temperature, humidity, and rainfall, all of which significantly influence decomposition rates and arthropod activity. For example, accelerated decomposition during hot summers contrasts sharply with slower rates observed in cooler seasons. Such variability makes it difficult to establish consistent baselines for PMI estimation and arthropod succession patterns [104,105]. Additionally, microclimatic factors, such as whether a cadaver is in sunlight or shade, introduce further inconsistencies into data collection.

Another challenge is the inherent bias in arthropod sampling methods, which tend to favor larger, more conspicuous species such as blow flies and flesh flies, while underrepresenting smaller or less obvious taxa like mites and beetles. Sampling bias may result from the use of specific trap designs, collection techniques, or human error in species identification, which can skew data and obscure the ecological roles of understudied arthropods in decomposition [40,100]. This challenge is exacerbated by the lack of standardized protocols for sampling and analyzing microarthropods in forensic contexts.

The final challenge is acknowledging that FARF has been founded on the principles of forensic anthropology. Disciplines such as FE are peripheral to this main research focus at FARF. Although an awareness of forensic entomology exists, most of the research is based on a holistic approach to taphonomy with insect activity only as a contributor to decomposition. Many students embarking on research at FARF have little or no knowledge of entomology, with most having only basic training in anthropology. Misfortunately, these HTFs are almost always associated with anthropology groups with only irregular links to entomology departments (Dadour, pers comm, 2025).

Logistical constraints also affect the scope and reliability of research. Longitudinal studies that observe decomposition over extended periods are often difficult to sustain due to limited funding or resources. This can lead to incomplete datasets, particularly during slower decomposition phases in cooler months [142]. Moreover, maintaining consistent monitoring of experimental setups, such as recording environmental data or managing trapping systems, adds another layer of complexity, with errors potentially compromising data quality.

Lastly, while microbial activity and acarology are increasingly recognized as important components of decomposition, these areas remain underrepresented in forensic research. Existing methodologies often fail to fully integrate microbial and acarological data with traditional entomological findings, resulting in an incomplete understanding of the decomposition process. The absence of standardized methods for incorporating microbial and acarological insights further limits their forensic application [105,140].

### 4.6. Future Research Directions

A significant gap in current forensic research is the underrepresentation of micro arthropods such as mites. While larger arthropods like blow flies and beetles have been studied in the facility, the ecological roles of smaller taxa such as acari remain poorly understood, particularly in conditions where larger scavengers are less active. These microarthropods may provide valuable forensic indicators, but their potential has yet to be fully explored [40,100]. Additionally, taxonomic expertise is required for accurate identification of forensically significant insects since this is often lacking in forensic research at FARF, creating gaps in data collection and interpretation.

As mentioned, there is a lack of longitudinal studies that capture the full range of environmental and ecological variables affecting decomposition. Many studies are short term and fail to account for seasonal or year-to-year variations, which are critical for establishing robust decomposition models. The semi-arid subtropical climate of FARF offers unique opportunities for long term studies that could fill this void [110,140].

Similarly, the underutilization of advanced molecular tools, such as microbial DNA sequencing and isotopic analyses, represents a missed opportunity to refine PMI estimation methods and uncover hidden ecological interactions [140]. Future research should prioritize the inclusion of micro-arthropods in forensic studies, with an emphasis on developing standardized sampling and identification protocols. Advances in molecular techniques, such as DNA barcoding, could facilitate the identification of these smaller taxa and shed light on their ecological roles in decomposition. Integrating these insights into forensic models would enhance the precision of PMI estimation.

Finally, emerging technologies such as remote sensing, machine learning and advanced imaging offer exciting opportunities to revolutionize forensic taphonomy [30]. These tools could enable real–time monitoring of decomposition sites, enhance the resolution of data collection and uncover patterns that are not apparent through conventional methods. By embracing these innovations, FARF and other facilities can continue to advance the field of forensic science and provide more reliable tools for investigations [100].

## 5. Conclusions

The present systematic review represents the first comprehensive synthesis of the scholarly outputs that have arisen from FARF, consisting of 15 studies that advance forensic entomology and human decomposition research. This analysis has highlighted diverse thematic areas which have covered PMI estimation, arthropod behavior, taxonomy, microbial interaction with arthropods and decomposition scenarios, all of which demonstrate the pivotal role of FARF in forensic science.

This review has systematically consolidated the contribution of forensic entomology and what it has delivered to the science of taphonomy of human remains at FARF. The intention of this review is to emphasize the understanding of the research undertaken in FE over the last 17 years at FARF and to provide the TXST Anthropology group with some thoughtful insights into how they might like to conduct this type of research into the future.

## Figures and Tables

**Table 1:** List of operational Human Taphonomic Facilities.

Country	Institution	Name—Acronym	Est.	Environment
USA	University of Tennessee, Knoxville	Anthropology Research Facility (ARF)	1981	Temperate, without dry season and hot summers
	Western Carolina University, Cullowhee	Forensic Osteology Research Station (FOREST)	2007	Temperate, without dry season and hot summers
	Texas State University, San Marcos (Freeman Ranch)	Forensic Anthropology Research Facility (FARF)	2008	Temperate, without dry season and hot summers
	Sam Houston State University, Texas	Southwest Texas Applied Forensic Science Facility (STAFS)	2008	Temperate, without dry season and hot summers
	Southern Illinois University, Carbondale	Complex for Forensic Anthropology Research (CFAR)	2010	Temperate, without dry season and warm summers
	Colorado Mesa University, Grand Junction	Forensic Investigation Research Station (FIRS)	2012	Arid, steppe and cold.
	University of Southern Florida, Tampa	USF Facility for Outdoor Research and Training (FORT)	2016	Subtropical, wet and dry season
	University of Tennessee, Oakridge	Cumberland Forest Decomposition Center (CFDC)	2016	Temperate, without dry season and hot summers
	Northern Michigan University, Marquette	Forensic Research Outdoor Station (FROST)	2017	Cold, dry winters
	Florida gulf coast University, Florida	Forensics Institute for Research, Security and Tactics (FIRST)	2017	Subtropical, mild winters, humid summers
	Louisiana State University, Baton Rouge	Forensic Taphonomy and Experimental Research Facility (FTERF)	2018	Subtropical, mild winters, hot humid summers
	George Mason University, Manassas, Virginia	Forensic Science Research and Training Lab (FSRTL)	2021	Humid subtropical and warm summers
Australia	University Technology Sydney	Australian Facility for Taphonomic and Experimental Research (AFTER)	2016	Temperate, without dry season and hot summer
Netherlands	Amsterdam UMC (University Medical Centers)	Amsterdam Research Initiative for Sub-surface Taphonomy and Anthropology (ARISTA)	2018	Temperate, without dry season and warm summer
Canada	Université du Québec à Trois-Rivières	Recherche en Sciences Thanatologiques [Experimentales et Sociales], (REST[ES])	2020	Humid, continental, cold winters, hot summers.

**Table 2:** Arthropod-related published records and dissertations involving Texas State University’s forensic anthropology research facility donated human remains.

Author(s)	Year	Type of Work	Major Findings and Contributions
Owings et al. [99]	2014	Published	Genetic variation among *Cochliomyia macellaria* (Fabricius) developmental relative to temperature
Pechal et al. [40]	2015		Observed unusual arthropod activity of Orthoptera, Isopoda and Hymenoptera on human remains
O’Connor et al. [41]			Conducted taxonomic revision of mites *Myianoetus muscarum* (Linnaeus) associated with human decomposition
Mohr and Tomberlin [100]			Prediction of TOP using adult blow fly (Diptera: Calliphoridae) carcass attendance
Singh et al. [45]	2018		Human cadaver decomposition has spatiotemporal effect on bacterial and arthropod communities
Corrêa et al. [101]	2021		Larval aggregation of *Dermestes maculatus* (De Geer) and *D. caninus* (Germar) development
Cuttiford et al. [102]	2021		BSF development is inconsistent for estimating TOP
Hyder [103]	2007	Dissertation	Containers and shading slow decomposition
Phalen [104]	2013		Body clothing slows decomposition
McDaneld [105]	2016		Tarp-covered remains have a higher decomposition rate due to higher insect activity compared to uncovered ones
George [106]	2017		Examines the effect of larval secretions and excretions on food source selection by *D. maculatus*
Cuttiford [107]			DD models need validation for *C. macellaria* to improve PMI accuracy
Spaulding [108]	2020		Shallow burials slow decomposition
Giacomello [109]	2022		Higher fat mass slow down decomposition
Young [110]	2023		Less pre colonization slows decomposition

**Table 3:** An overview of the arthropod taxa recovered or reported at FARF, 2007–2023. [“N” denotes missing, unspecified or unreported taxonomic or descriptive information as presented in the original source material (dissertation or publication)].

Year of Record	Literature Source	Taxon— Scientific Name	Common Name— Descriptor	Higher Level Taxonomic Classification	Family
2007	Hyder [103]	N	Blow flies	Diptera	Calliphoridae
			Flesh flies	Diptera	Sarcophagidae
			Black soldier flies	Diptera	Stratiomyidae
			Larval masses	Diptera	N
			–	Coleoptera	Dermestidae
				Hymenoptera	Formicidae
2013	Phalen [104]	*Chrysomya rufifacies* (Macquart)	Hairy maggot blow fly	Diptera	Calliphoridae
		*C. macellaria*	Secondary screwworm	Diptera	Calliphoridae
		N	Adult Flies	Diptera	N
			Gnats	Diptera	
			Biting Ants	Hymenoptera	Formicidae
		*Solenopsis* spp.	Fire Ants	Hymenoptera	Formicidae
		*Dorymyrmex pyramicus* Roger	Pyramid ants	Hymenoptera	Formicidae
		N	Beetles	Coleoptera	N
		*Dermestes lardarius* Linnaeus	N	Coleoptera	Dermestidae
		N	Spiders	Araneae	N
			Praying mantis	Mantodea	
			Cockroaches	Blattodea	
		N	Katydids	Orthoptera	Tettigoniidae
			Mosquitoes	Diptera	N
2014	Owings et al. [99]	*C. macellaria*	Secondary screwworm	Diptera	Calliphoridae
2015	Mohr and Tomberlin [100]	*Phormia regina* Meigen	Black blow fly	Diptera	Calliphoridae
		*C. rufifacies*	Hairy maggot blow fly	Diptera	Calliphoridae
		*Calliphora vicina* (Robineau-Desvoidy)	Blue bottle fly	Diptera	Calliphoridae
		*C. macellaria*	Secondary screwworm	Diptera	Calliphoridae
2015	O’Connor et al. [41]	*Myianoetus muscarum* (Linnaeus)	Mites	Acari	Histiostomatidae
2015	Pechal et al. [40]	*Pediodectes haldemani* Girard	Katydid	Orthoptera	Tettigoniidae
		*Armadillidium* cf. *vulgare* (Latreille)	Pill bug	Isopoda	Armadillidiidae
		*Solenopsis invicta* Buren	Red Imported Fire Ant	Hymenoptera	Formicidae
2016	McDaneld [105]	N	Blow fly larvae	Diptera	Calliphoridae
2017	George [106]	*D. maculatus*	N	Coleoptera	Dermestidae
2017	Cuttiford [107]	*C. macellaria*	Secondary screwworm	Diptera	Calliphoridae
2018	Singh et al. [45]	N	N	Hymenoptera	Formicidae
				Diptera	N
				Coleoptera	Staphylinidae
				Coleoptera	Carabidae
				Collembola	Isotomidae
				Acari	Acaridae
				Mesostigmata	Uropodidae
				Oribatida	Pthiricaridae
				Acari	Prostigmata
2020	Spaulding [108]	*Hermetia illucens* Linnaeus	Black soldier fly	Diptera	Stratiomyidae
		N	Phoretic mites	Acari	N
			Ants	Hymenoptera	Formicidae
			Larvae	Diptera	–
2021	Cuttiford et al. [102]	*H. illucens*	Black soldier fly	Diptera	Stratiomyidae
	Corrêa et al. [101]	*D. maculatus*	N	Coleoptera	Dermestidae
		*D. caninus*			
2022	Giacomello [109]	N	Blow fly	Diptera	Calliphoridae
2023	Young [110]	*Sarcophaga* spp.	Flesh flies	Diptera	Sarcophagidae
		*Lucilia* spp.	Green bottle flies	Diptera	Calliphoridae
		*Solenopsis* spp.	Fire ants	Hymenoptera	Formicidae
		N	Larval masses	Diptera	N

## Data Availability

The original contributions presented in this study are included in the article. Further inquiries can be directed to the corresponding author.
